# Prognostic Role of Squamous Cell Carcinoma Antigen in Cervical Cancer: A Meta-analysis

**DOI:** 10.1155/2019/6710352

**Published:** 2019-06-02

**Authors:** Zhenhua Liu, Hongtai Shi

**Affiliations:** ^1^Department of Radiotherapy, Yancheng City No. 1 People's Hospital, 66 Renmin Street, Yancheng 224000, China; ^2^Department of Radiotherapy, The Third People's Hospital of Yancheng, 75 Juchang Street, Yancheng 224005, China

## Abstract

**Objective:**

To systematically evaluate the significance of squamous cell carcinoma antigen (SCC-Ag) in the prognosis of cervical cancer.

**Methods:**

Literature from Pubmed, Embase, and Cochrane Library was retrieved to collect all English literature on the correlation between SCC-Ag and cervical cancer prognosis, and the quality of literature collected was assessed based on evaluation criteria. The heterogeneity, sensitivity, and specificity were detected using the StataSE12.0 software, and the correlation between SCC-Ag and cervical cancer prognosis as the effect variables was assessed using the hazard ratio (HR) and 95% confidence interval (CI). Moreover, the forest map and funnel plot were drawn.

**Results:**

A total of 17 articles meeting the inclusion criteria were selected. The high expression of SCC-Ag was significantly correlated with the poor prognosis of cervical cancer (HR = 2.22, 95% CI = 1.38 − 3.57, *P* = 0.002). The disease-free survival (DFS) was higher in low SCC-Ag expression patients than in high SCC-Ag expression patients (HR = 2.17, 95% CI = 1.84 − 2.57, *P* < 0.001). The progression-free survival (PFS) was inferior in patients with a high SCC-Ag expression (HR = 2.70, 95% CI = 1.11 − 6.53, *P* = 0.028).

**Conclusion:**

SCC-Ag is an important prognostic factor for cervical cancer, and its high expression is significantly correlated with a poor prognosis of the disease.

## 1. Introduction

Cervical cancer is the third most common cancer in females around the world and the fourth leading cause of cancer death [1]. Compared with developed countries, developing countries have a significantly higher incidence rate of cervical cancer, which is related to the intensity of cervical cancer screening [1]. More than 270,000 women die of cervical cancer every year, more than 85% of whom die in low- and middle-income countries [1]. Due to insufficient resources, cervical cancer prevention and control cannot achieve high coverage in these countries [2]. The early symptoms of cervical cancer are not obvious, so patients are often diagnosed in the advanced stages, which directly affects the quality of life and prognosis of patients. The common pathological types of cervical cancer are squamous cell carcinoma, adenocarcinoma, and adenosquamous carcinoma. Squamous cell carcinoma is the most common. Squamous cell carcinoma antigen (SCC-Ag) is an early tumour marker for diagnosing cervical cancer and monitoring responses to treatment in the event of relapse. It is convenient to detect, has good specificity, and is widely used in clinical practice. Studies have demonstrated that an increase in SCC-Ag concentration precedes clinical manifestations and can be used as an independent risk factor [3]. Moreover, SCC-Ag is commonly used in the detection of lung cancer, oesophageal cancer, and head and neck cancer. In the diagnosis and detecting recurrence of cervical cancer, SCC-Ag has been clearly validated. In terms of the prognosis, many studies argued that it was an independent prognostic factor. The clinical stage, depth of invasion, tumour size, and lymph node metastasis of SCC have enormous significance in the prognosis [4]. By continuous monitoring of SCC-Ag, early SCC recurrence may be detected and appropriate remedial treatment can be prescribed for early intervention, thereby improving the quality of life and prolonging survival [5]. However, there have been studies showing the different prognostic values of SSC-Ag in cervical cancer. Not all patients with recurrent cervical cancer have sustained SCC-Ag during follow-up, and some have SCC-Ag during disease progression. No specific changes have been identified that can guide the development of clinical treatment strategies for patients. This study comprehensively searched the literature on the correlation between SCC-Ag and cervical cancer prognosis and systematically analyzed appropriate publications by meta-analysis.

## 2. Materials and Methods

### 2.1. Literature Retrieval

The literature up to June 2018 was independently searched by two researchers in the library records. Pubmed, Embase, and Cochrane Library entries were retrieved. English index words included SCC-Ag, squamous cell carcinoma antigen, serum squamous carcinoma antigen, tumour, cancer, carcinoma, neoplasm, prognostic, prognosis, survival, carcinoma of uterine cervix, and cervical cancer.

### 2.2. Inclusion and Exclusion Criteria of Literature

Publication inclusion criteria are as follows: (1) studies based on cervical cancer patients; (2) published epidemiological studies, such as case-control studies and line-up studies; (3) studies evaluating the diagnostic value of tumour markers for cervical cancer; (4) positive values or HR and 95% CI of the case group and the control group were clearly reported, or the corresponding four-table data were provided; (5) research methods and scope were similar, and detection methods were the same; (6) the relationship between SCC-Ag expression and prognosis of cervical cancer was reported; and (7) the data and information were complete.

Publication exclusion criteria are as follows: (1) publications containing the pathological type of noncervical cancer; (2) cases that had no clear diagnostic criteria; (3) reviews or conference minutes; (4) the number of cases was less than 10; (5) publications that did not provide credible case and control sources; (6) lack of raw data to calculate the HR, 95% CI, and *P* value; (7) inconsistent publications; and (8) publications that lacked the results or controversial publications.

### 2.3. Literature Screening

Two researchers independently screened the literature according to the inclusion criteria. If there were differences or disputes in the process, they reached an agreement through discussion and study. The first step was to exclude irrelevant articles from the topic and abstract reading; the second step was to exclude publications without reference values; the third step was to exclude articles with no integrity and accuracy data; and, finally, the articles meeting the inclusion and exclusion criteria after screening were obtained.

### 2.4. Data Extraction

Two researchers independently screened the literature, extracted and cross-checked the data, searched the relevant publications according to the search strategy, screened the appropriate publications by looking through their titles and abstracts, and read the full text to determine whether the inclusion criteria were met. The information to be extracted mainly included the first author's name and nationality, publication period, number of patients, pathological stage, age, follow-up time, test samples, test methods, cut-off values, analysis methods of the survival rate, and survival analysis.

### 2.5. Statistical Analysis

The correlation between SCC-Ag expression and cervical cancer prognosis as the effect variables was evaluated by the HR value and 95% CI. If the HR and 95% CI were described in the publication, they were extracted directly. If not described, they were calculated based on available data. Data for the Kaplan-Meier survival curve were obtained by the Engauge software. A meta-analytical method was used to calculate and merge the results of the publications that met the inclusion criteria, and the Stata12.0 software was used to draw the forest map. If HR > 1, patients with a high SCC-Ag expression had a worse prognosis; if HR < 1, patients with a high SCC-Ag expression had a better prognosis; and if HR = 1, the high expression of SCC-Ag was not related to the prognosis of patients. The heterogeneity of this study was investigated by *I*^2^ and *P*. If *I*^2^ > 50% and *P* < 0.05, the heterogeneity among studies was strong and the data were combined by a random effects model; if *I*^2^ < 50% and *P* > 0.05, there was no heterogeneity among studies and the data were combined using a fixed effects model. The Stata12.0 software was also used to draw the funnel plot to find potential publication bias.

## 3. Results

### 3.1. Literature Retrieval Results

**Figure 1:**
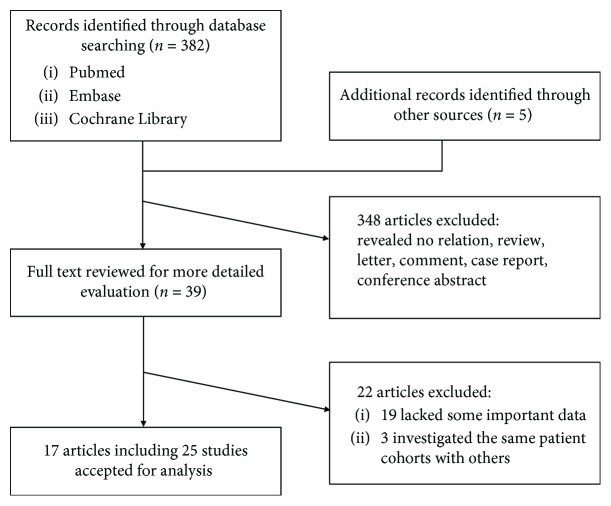
Flow diagram of the study selection process.

A total of 387 publications related to SCC-Ag expression and the prognosis of cervical cancer were retrieved. Of these, 348 publications unrelated to the study were excluded by carefully reading the title and abstract, and the remaining 39 publications were further examined by screening the full text. Finally, 17 publications were studied, including 4 that simultaneously analyzed overall survival (OS) and progression-free survival (PFS) as prognostic indicators, 4 that analyzed OS and disease-free survival (DFS) as prognostic indicators, 5 studied with DFS alone, 3 articles with the most endpoints of OS alone, and 1 studied with PFS alone (Figure 1) [6–22]. The publication period of the literature was between 2003 and 2017. A total of 3308 patients from different countries were included. Patients mostly had pathological stages II-III. The average age ranged from 42.7 to 68 years. The minimum follow-up time was 34 months, and 60 months was the longest follow-up period. The cut-off value of each article was selected according to the research needs. The test samples extracted from the patients' serum in 18 articles were analyzed by ELISA. The basic characteristics of the included literature are shown in Table 1.

### 3.2. Meta-analysis

**Figure 2:**
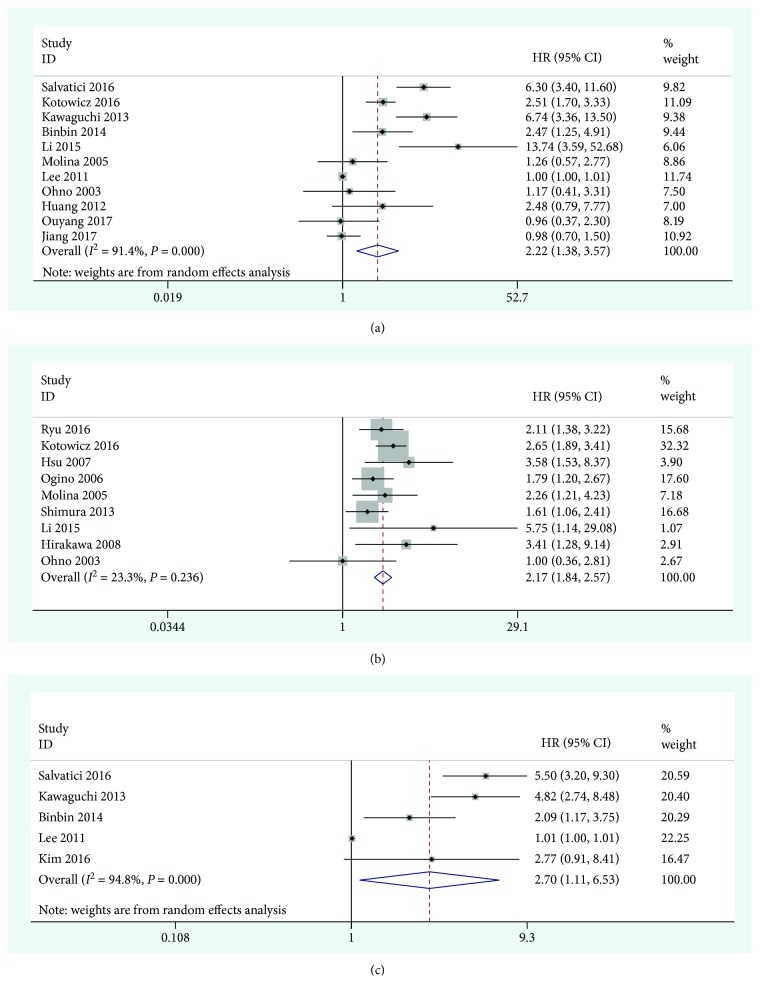
Forest plot of the relationship between high SCC-Ag and OS (a), DFS (b), and PFS (c) in cervical cancer.

A total of 11 publications including 2441 patients provided the data for the OS analysis. Meta-analysis using the Stata12.0 software showed statistically significant heterogeneity in the study of OS (*I*^2^ = 91.4%, *P* < 0.001). The random effects model was used to combine the HRs. The pooled results showed that the high expression of SCC-Ag was significantly associated with the poor prognosis of cervical cancer (combined HR = 2.22, 95% CI = 1.38 − 3.57, *P* = 0.002). The forest graph is shown in Figure 2(a). In addition, the studies evaluating DFS and PFS were summarized and analyzed. The results showed that there was no statistical heterogeneity in the study of DFS (*I*^2^ = 23.3%, *P* = 0.236), and the combined effects model was used to merge the HRs (HR = 2.17, 95% CI = 1.84 − 2.57, *P* < 0.001) (Figure 2(b)). There was significant heterogeneity in the study of PFS (*I*^2^ = 94.8%, *P* < 0.001), and HR was combined using a random effects model (HR = 2.70, 95% CI = 1.11 − 6.53, *P* = 0.028) (Figure 2(c)). The cause of heterogeneity may be related to race and the small number of articles included.

### 3.3. Subgroup Analysis

**Figure 3:**
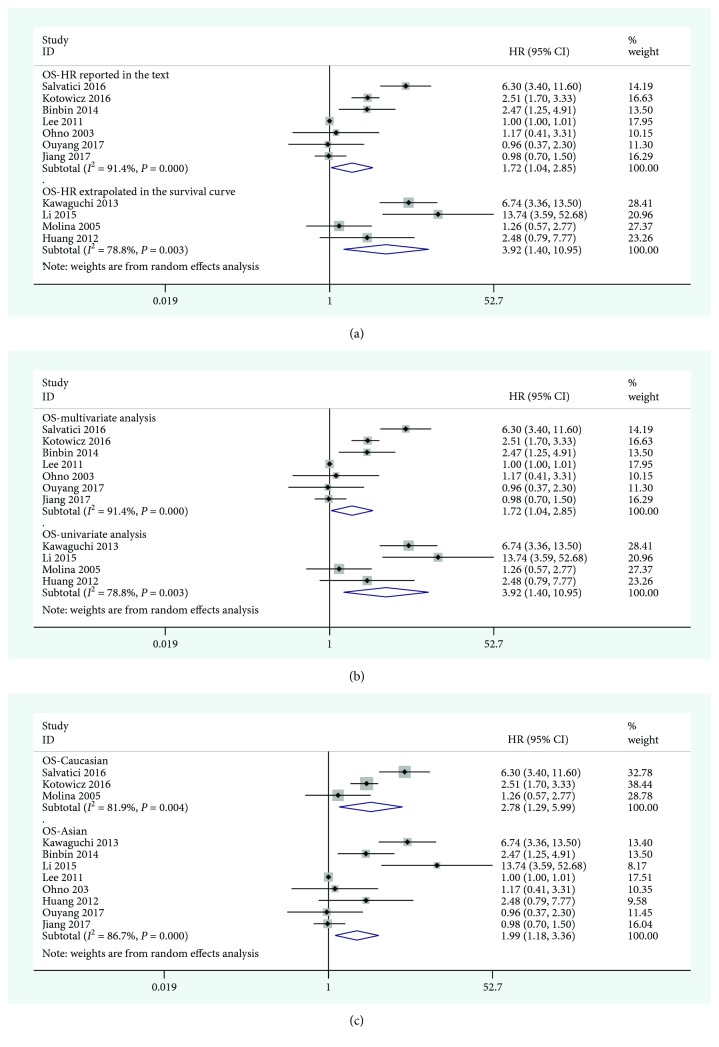
Forest plot of the relationship between high SCC-Ag and OS in the HR obtain method subgroup (a), the analysis-type subgroup (b), and the ethnicity subgroup (c).

**Figure 4:**
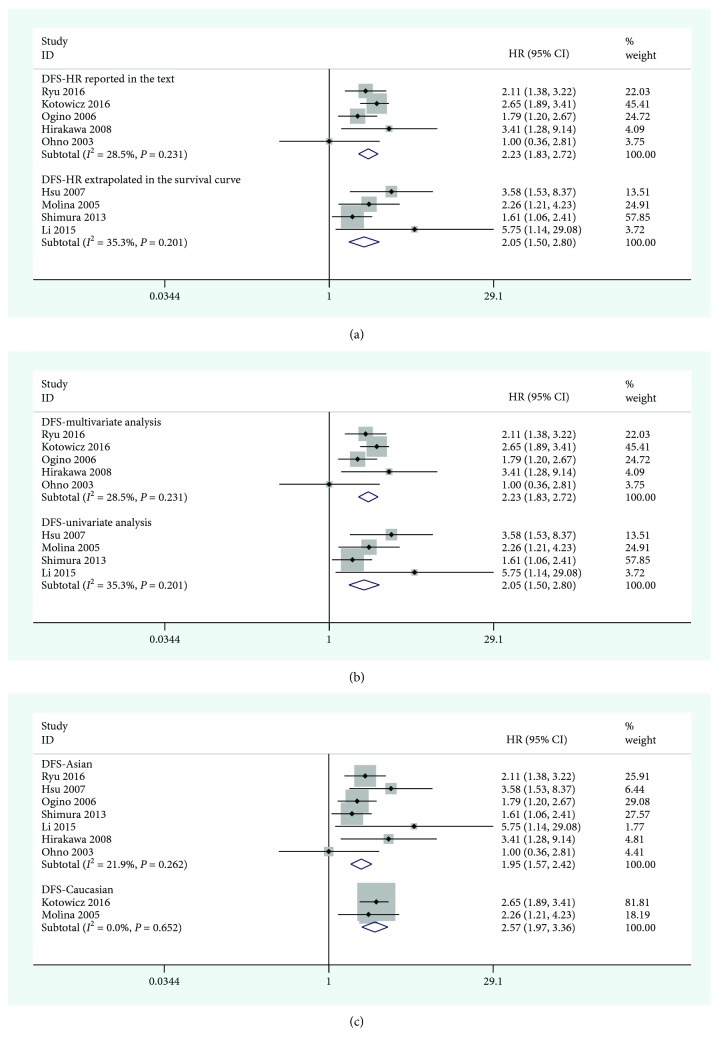
Forest plot of the relationship between high SCC-Ag and DFS in the HR obtain method subgroup (a), the analysis-type subgroup (b), and the ethnicity subgroup (c).

According to the clinical characteristics and the results of the meta-analysis, the studies providing OS (Figure 3) and DFS (Figure 4) data were separately analyzed in subgroups. The results are shown in Table 2. In the ethnic subgroup analysis, the high expression of SCC-Ag was a poor prognostic factor for cervical cancer. In the other subgroup analyses, such as the data acquisition group and the factor analysis group, there were also statistical significance. In the prognostic OS subgroup analysis, the results for the HR reported in the text group were HR = 1.72 (95% CI = 1.04 − 2.85, *P* = 0.038) and those for the HR-extrapolated group were HR = 3.92 (95% CI = 1.40 − 10.95, *P* = 0.009). In the racial analysis, the results for East Asian were HR = 1.99 (95% CI = 1.18 − 3.36, *P* = 0.009) and those for Caucasian race were HR = 2.78 (95% CI = 1.29 − 5.99, *P* = 0.010). Multivariate analysis of prognostic DFS subgroups resulted in HR = 2.23 (95% CI = 1.83 − 2.72, *P* < 0.001), whereas the univariate analysis results were HR = 2.05 (95% CI = 1.50 − 2.80, *P* < 0.001); the results for East Asian were HR = 1.95 (95% CI = 1.57 − 2.42, *P* < 0.001), and those for Caucasian race were HR = 2.57 (95% CI = 1.97 − 3.36, *P* < 0.001).

### 3.4. Publication Bias

**Figure 5:**
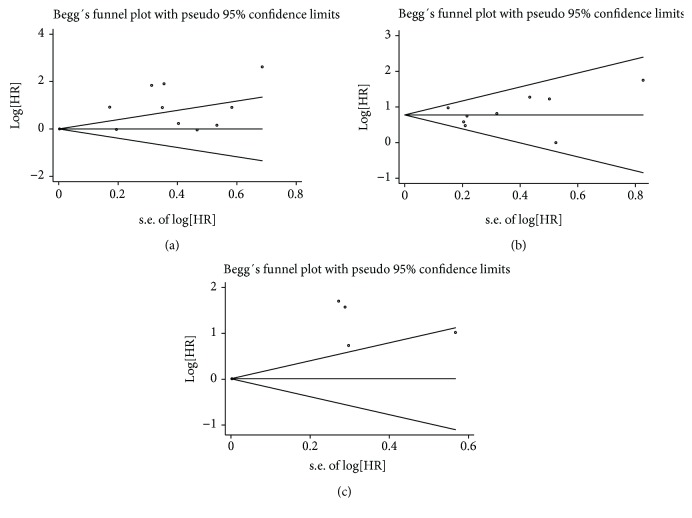
Sensitivity analysis for meta-analysis between high SCC-Ag and OS (a), DFS (b), and PFS (c) in cervical cancer.

The publication bias was determined by Egger's test and Begg's test, and the resulting funnel plot was asymmetrical, as shown in Figure 5, indicating that the study had a publication bias.

## 4. Discussion

SCC-Ag is a glycoprotein, a subtype of the tumour-associated antigen TA-4, with a molecular weight of approximately 45 kDa. It is a clinically important tumour marker. Kato and Torigoe invented a radioimmunoassay for simple and convenient detection [23]. It has advantages over imaging tests, such as B-ultrasound, CT, and MRI. SCC-Ag can act as a serine/cysteine protease inhibitor with components involved in the degradation of extracellular matrices and tumour invasion and metastasis [24]. SCC-Ag is expressed in a normal squamous epithelium and can be used clinically for the diagnosis and monitoring responses to treatment in the event of cervical, lung, oesophageal, and head and neck cancer but especially in the diagnosis of cervical cancer, with specificity higher than 80%. Therefore, it has been widely used in recent years and is recognized as a highly reliable serum cervical cancer tumour marker [25]. Many scholars have conducted research on this tumour marker. For example, Cox analysis indicated that SCC-Ag is an independent prognostic factor for OS and DFS, as shown by Kotowicz et al. [6]. Ryu et al. showed by single-factor and multivariate analyses that the high SCC-Ag expression and poor DFS were statistically significantly correlated [8]. However, some studies have demonstrated that SCC-Ag is not a completely reliable prognostic factor. For example, a previous study indicated that SCC-Ag was statistically significant in the univariate analysis of the DFS prognosis but not in the multivariate analysis [14]. Additionally, in the study by Molina et al., Kaplan-Meier survival curves showed that the high expression of SCC-Ag did not indicate poor OS [19]. In addition, SCC-Ag combined with other tumour markers, such as CEA, CA 19-9, and CYFRA21-1, had significance in the diagnosis and prognosis of squamous cell carcinoma. At the same time, some studies have noted the value of SCC-Ag and IL-6, VEGF, and TNF*α* in diagnosis [26]. Cervical cancer is mainly treated with surgery, radiotherapy, and chemotherapy. Synchronous chemoradiotherapy is often used in patients with advanced disease. SCC-Ag can also be used as an independent predictor of OS, disease-specific survival, and distant metastasis during treatment [27, 28]. In 1998, Maruo et al. showed a positive correlation between the EGF receptor and SCC antigen expression levels. Thus, an increase in the level of SCC antigen can indicate the amount of cell proliferation that occurs in cervical cancer [29]. Serum SCC-Ag indicates a certain tumour burden in patients with cervical cancer. As the disease progresses to the later pathological stages of cervical cancer, the depth of infiltration is deeper, the tumour volume is larger, and SCC-Ag enters the tumour lymphatics. This increases the likelihood that the tumour cells will enter the bloodstream, and as more cancer antigens are produced and released into serum, the higher levels of SCC-Ag are detected in the venous blood of patients with cervical cancer. This antigen is involved in the development of tumours and thus is related to the treatment and prognosis of tumours, with a certain regulatory effect in the process of apoptosis.

In the meta-analysis, it was found that the high expression of SCC-Ag was significantly associated with the poor OS of patients with cervical cancer (HR = 2.73, 95% CI = 1.48 − 5.05, *P* = 0.001). However, the cut-off values used in these studies were all different. The most used cut-off value was 1.5 ng/mL, which may be a criterion for predicting prognosis, but it needs to be further verified, and there may be errors in some data extraction processes. In addition, the follow-up time of each study was different, and some patients were lost to follow-up or were false positives. Moreover, the literature included a retrospective study that may affect the accuracy and authenticity of the data. Therefore, the reasons for the obvious heterogeneity of this meta-analysis may be the inclusion of English literature, the small number of included publications, tumour stage, cut-off value, follow-up time, and other factors. Finally, although this is the only marker assessed in the study, we should realize its limitations that other factors may be more sensitive. Future research needs to focus on the combination between SCC and other important factors of cervical cancer. In the clinical treatment of cervical cancer, it is necessary to accurately diagnose the patient, analyze the patient's pathological stage and lymphatic metastasis, dynamically observe the changes in the patient's SCC-Ag, and implement remedial measures in advance to improve the patient's quality of life and reduce their pain.

## 5. Conclusion

This meta-analysis summarizes all studies of SCC-Ag and cervical cancer prognosis, indicating that the high expression of SCC-Ag is significantly correlated with poor OS in cervical cancer. Considering the limitations of the study, further prospective and large-sample studies are needed to confirm the prognostic value of SCC-Ag in cervical cancer and to provide evidence for a better investigation of treatment strategies.

## Figures and Tables

**Table 1:** Main characteristics of all studies included in the meta-analysis.

Author	Country	Year	Sample number	FIGO stage I/II/III/IV	Age	Follow-up months	Detected sample	Test way	Cut-off value (ng/mL)	Multivariate analysis	Survival analysis
Ouyang et al. [20]	China	2017	155	101/54/0/0	Median 47	Over 60	Serum	ELISA	1.5	Yes	OS
Jiang et al. [21]	China	2017	182	IB-IIA153/IIB-IIIB29	Mean 53	Over 60	Serum	ELISA	4	Yes	OS
Kim et al. [22]	Korea	2016	48	9/28/6/5	Median 52	Median 34	Serum	ELISA	2	Yes	PFS
Salvatici et al. [7]	Italy	2016	197	158/39/0/0	Median 43.9	Over 60	Serum	ELISA	1.5	Yes	OS, PFS
Ryu et al. [8]	Korea	2016	154	53/84/9/8	Median 59	Median 41	Serum	ELISA	1.86	Yes	DFS
Kotowicz et al. [6]	Poland	2016	138	38/40/57/3	Median 54	Over 60	Serum	ELISA	1.6/1.8	Yes	OS, DFS
Li et al. [9]	China	2015	286	IB1-IIA135/IIB-IIIB151	Median 45	Over 60	Serum	ELISA	3.5	No	OS, DFS
Binbin et al. [10]	China	2014	172	0/119/50/3	Median 49	Median 54.5	Serum	ELISA	3	Yes	OS, PFS
Shimura et al. [11]	Japan	2013	167	22/66/45/34	Median 58	Over 60	Serum	ELISA	2	No	DFS
Kawaguchi et al. [12]	Japan	2013	116	IIB33/IIIA6/IIIB64/IVA13	Median 68	Median 58.6	Serum	ELISA	1.15	No	OS, PFS
Hirakawa et al. [13]	Japan	2008	108	IB-2/II-57/III-46/IV-3	Median 50	Median 48	Serum	ELISA	1.5	Yes	DFS
Hsu et al. [14]	Taiwan	2007	38	IB-32/IIA-6	Median 50.8	Median 29.8	Serum	ELISA	NR	No	DFS
Qgino et al. [15]	Japan	2006	352	IIB-99/III-239/IVA-14	Median 61	Median 53	Serum	ELISA	6	Yes	DFS
Molina et al. [19]	Spain	2005	156	47/65/39/5	NR	Median 44	Serum	ELISA	2	No	OS, DFS
Huang et al. [16]	Taiwan	2012	188	22/125/37/4	Median 56.5	Median 58.2	Serum	ELISA	10	No	OS
Lee et al. [17]	Korea	2011	788	505/219/50/14	Median 51	Median 53.4	Serum	ELISA	1.6	Yes	OS, PFS
Ohno et al. [18]	Japan	2003	63	2/21/29/11	Median 66	Over 48	Serum	ELISA	1.5	Yes	OS, DFS

NR: no report; OS: overall survival; PFS: progression-free survival; DFS: disease-free survival.

**Table 2:** The pooled associations between different situations of SCC-Ag expression and the prognosis of patients with cervical cancer.

Outcome subgroup	No. of patients	No. of studies	HR (95% CI)	*P* value	Heterogeneity	
					*I* ^2^	*P*
OS	2441	11	2.22 (1.38-3.57)^b^	0.002	91.4	<0.001
HR obtain method						
Reported in the text	1918	7	1.72 (1.04-2.85)^b^	0.038^∗^	91.4	<0.001
Data extrapolated	523	4	3.92 (1.40-10.95)^b^	0.009	78.8	0.003
Analysis type						
Multivariate	1918	7	1.72 (1.04-2.85)^b^	0.038^∗^	91.4	<0.001
Univariate	523	4	3.92 (1.40-10.95)^b^	0.009	78.8	0.003
Ethnicity						
Asian	1950	8	1.99 (1.18-3.36)^b^	0.009^∗^	96.7	<0.001
Caucasian	491	3	2.78 (1.29-5.99)^b^	0.010^∗^	81.9	0.004
DFS	1462	9	2.17 (1.84-2.57)^a^	<0.001	23.3	0.236
HR obtain method						
Reported in the text	815	5	2.23 (1.83-2.72)^a^	<0.001	28.5	0.231
Data extrapolated	647	4	2.05 (1.50-2.80)^a^	<0.001	35.3	0.201
Analysis type						
Multivariate	815	5	2.23 (1.83-2.72)^a^	<0.001	28.5	0.231
Univariate	647	4	2.05 (1.50-2.80)^a^	<0.001	35.3	0.201
Ethnicity						
Asian	1348	7	1.95 (1.57-2.42)^a^	<0.001	21.9	0.262
Caucasian	294	2	2.57 (1.97-3.36)^a^	<0.001	0	0.652
PFS	1321	5	2.70 (1.11-6.53)^b^	0.028	94.8	<0.001

OS: overall survival; DFS: disease-free survival; PFS: progression-free survival; HR: hazard ratio; CI: confidence interval. ^a^Fixed effects model. ^b^Random effects model.

## Data Availability

The data used to support the findings of this study are available from the corresponding author upon request.
